# Diagnostic value of immunoglobulin κ light chain gene rearrangement analysis in B-cell lymphomas

**DOI:** 10.3892/ijo.2014.2790

**Published:** 2014-12-09

**Authors:** IRA KOKOVIC, BARBARA JEZERSEK NOVAKOVIC, SRDJAN NOVAKOVIC

**Affiliations:** 1 Department of Molecular Diagnostics, Institute of Oncology Ljubljana, Ljubljana, Slovenia; 2 Division of Medical Oncology, Institute of Oncology Ljubljana, Ljubljana, Slovenia

**Keywords:** BIOMED-2, clonality analysis, B-cell lymphomas, *IGH* rearrangement, IGK rearrangement

## Abstract

Analysis of the immunoglobulin κ light chain (*IGK*) gene is an alternative method for B-cell clonality assessment in the diagnosis of mature B-cell proliferations in which the detection of clonal immunoglobulin heavy chain (*IGH*) gene rearrangements fails. The aim of the present study was to evaluate the added value of standardized BIOMED-2 assay for the detection of clonal *IGK* gene rearrangements in the diagnostic setting of suspected B-cell lymphomas. With this purpose, 92 specimens from 80 patients with the final diagnosis of mature B-cell lymphoma (37 specimens), mature T-cell lymphoma (26 specimens) and reactive lymphoid proliferation (29 specimens) were analyzed for B-cell clonality. B-cell clonality analysis was performed using the BIOMED-2 IGH and IGK gene clonality assays. The determined sensitivity of the IGK assay was 67.6%, while the determined sensitivity of the IGH assay was 75.7%. The sensitivity of combined IGH+IGK assay was 81.1%. The determined specificity of the IGK assay was 96.2% in the group of T-cell lymphomas and 96.6% in the group of reactive lesions. The determined specificity of the IGH assay was 84.6% in the group of lymphomas and 86.2% in the group of reactive lesions. The comparison of GeneScan (GS) and heteroduplex pretreatment-polyacrylamide gel electrophoresis (HD-PAGE) methods for the analysis of *IGK* gene rearrangements showed a higher efficacy of GS analysis in a series of 27 B-cell lymphomas analyzed by both methods. In the present study, we demonstrated that by applying the combined IGH+IGK clonality assay the overall detection rate of B-cell clonality was increased by 5.4%. Thus, we confirmed the added value of the standardized BIOMED-2 IGK assay for assessment of B-cell clonality in suspected B-cell lymphomas with inconclusive clinical and cyto/histological diagnosis.

## Introduction

The assessment of B-cell clonality by analysis of immunoglobulin (Ig) gene rearrangements is an important tool in the diagnosis of suspected B-cell proliferations, for which the results of cyto/histopathological and immunophenotyping analysis are inconclusive (1–5). The immunoglobulin heavy chain gene (*IGH*) rearrangements have been the most frequent targets for clonality analysis by polymerase chain reaction (PCR) in mature B-cell proliferations (5). However, this method fails to detect clonal rearrangements of the *IGH* gene in a significant proportion of B-cell lymphomas. The most possible reason for these false negative results could be the process of somatic hypermutation (SHM) (4–8). SHM is a cellular mechanism by which the immune system adapts B cell receptors to recognize foreign antigens and to respond by production of specific immunoglobulins. The SHM process takes place in germinal center (GC) cells in the secondary lymphoid organs. As a result of SHM, variable (V_H_) and joining (J_H_) sequences of the rearranged VDJ exon of the *IGH* gene are altered by single-nucleotide mutations or small deletions or insertions of nucleotides. Thus, SHM can be responsible for preventing primer annealing, which leads to false-negative *IGH*-PCR results. The majority of B-cell lymphomas arise from GC and post-GC cells potentially carrying the risk of somatic mutations and lack of monoclonal *IGH*-PCR result (1–3,5).

The immunoglobulin light chain genes represent an alternative target for B-cell clonality assessment. It has been shown that detection of clonal rearrangements in the immunoglobulin κ light chain gene (*IGK*) can improve clonality detection rates in mature B-cell lymphomas that are heavily somatically mutated (4,5,9,10). During normal B-cell differentiation, rearrangements of the *IGK* gene start soon after the *IGH* gene rearrangements, and are followed by rearrangements of the immunoglobulin λ light chain gene (*IGL*). A functional *IGK* rearrangement produces Vκ-Jκ product, and generates an IGK^+^ B-cell. If a non-functional Vκ-Jκ product is generated, *IGK* allele is inactivated through recombination of the κ-deleting element (Kde). In a case of non-functional Vκ-Jκ rearrangements on both alleles, rearrangements of the IGL gene take place and generate an IGL^+^ B-cell. Thus, clonal Vκ-Jκ rearrangements should be detected in IGK^+^ B-cell lymphomas, and at least one clonal Kde rearrangement should be detected in IGL^+^ B-cell lymphomas (5,11,12).

The applicative value of the *IGK* gene rearrangement analysis in suspected B-cell lymphomas, particularly in cases of germinal center (GC) and post-GC lymphomas has been reported in a number of studies (4–8,13,14). In our previous study, we evaluated the utility of standardized BIOMED-2 clonality assay protocols for clonality analysis in a routine diagnostical setting of non-Hodgkin lymphomas (15). In the aforementioned study, we used only the assay protocol for the detection of clonal rearrangements in the *IGH* gene for assessment of B-cell clonality (15).

The aim of the present study was to evaluate the added value of standardized BIOMED-2 assay for detection of clonal *IGK* gene rearrangements in the diagnostic setting of suspected B-cell lymphomas using fresh and formalin-fixed diagnostic specimens.

## Materials and methods

### Study group

Ninety-two specimens from 80 patients submitted for routine diagnostics were evaluated in the present study. The study group included 37 specimens from 32 patients with B-cell lymphoma. Twenty-seven specimens were previously evaluated in our recently published study (15) and 10 specimens of suspected B-cell lymphoma were analyzed during routine diagnostic assessment from October through December 2013. In addition to B-cell lymphomas, 26 specimens of T-cell lymphomas (T-NHLs) and 29 specimens of reactive lymphoid proliferations were also included in the study. Different types of diagnostic samples were analyzed including 41 bone marrow (BM) aspirates, 25 fine-needle aspiration specimens (FNA), 22 formalin-fixed, paraffin-embedded tissue specimens (FFPE), 3 pleural fluid and 1 ascites.

All specimens were subjected to the cyto/histomorphological and immunophenotyping examination as well as to the PCR-based clonality analysis of B-cell populations during routine diagnostic assessment.

### Final diagnosis

The final diagnosis of each lymphoid proliferation was set upon careful evaluation of clinical, morphological, immunophenotyping and molecular data. The diagnosis of lymphoma subtype was made according to the WHO Classification of Tumours of Hematopoietic and Lymphoid Tissues (16).

### DNA isolation

DNA was isolated using commercial DNA isolation kits according to the manufacturers’ protocols. The QIAamp FFPE Tissue kit (Qiagen GmbH, Hilden, Germany) was used for FFPE tissue specimens and High Pure PCR Template Preparation kit (Roche Applied Science, Penzberg, Germany) was used for other types of specimens. The concentration and the purity of DNA (A_260nm_/A_280nm_) were determined using the NanoDrop spectrophotometer (Thermo Scientific, Wilmington, DE, USA).

### B-cell clonality analysis

#### BIOMED-2 clonality assays

B-cell clonality was assessed using the BIOMED-2 clonality assays-ABI Fluorescence Detection (IdentiClone; InVivo Scribe Technologies, San Diego, CA, USA) according to the manufacturer’s instructions. Rearrangements in the immunoglobulin heavy chain gene (*IGH*) were analyzed using the IdentiClone IGH Gene clonality assay and rearrangements in the immunoglobulin κ light chain gene (*IGK*) using the IdentiClone IGK Gene clonality assay.

The DNA quality was checked for all samples using the control gene PCR (Specimen Control Size Ladder Master Mix) and only samples yielding control gene PCR products of ≥ 300 bp were included in the study.

Based on the results of our previous study (15) the *IGH* clonality was evaluated with three IGH multiplex PCR reactions for detection of the complete rearrangements (V_H_-J_H_) in the *IGH* gene. The *IGK* clonality was evaluated with two IGK multiplex PCR reactions, one for the detection of the complete rearrangements (V_κ_-J_κ_) and one for the detection of Kde (κ deleting element) rearrangements in the *IGK* gene. Each run included monoclonal and polyclonal control DNAs for particular primer Master Mix, supplied with each BIOMED-2 clonality assay, and a contamination control (no template DNA in a reaction).

### Analysis of IGH and IGK amplification products

The fluorescently labeled PCR products were detected by capillary gel electrophoresis using the ABI 3500 Genetic Analyzer (Applied Biosystems, Foster City, CA, USA) and analyzed by fragment analysis (GeneScan), which discriminates amplification products according to their size (in bp). Fragment analysis was performed using GeneScan™ 600LIZ^®^ Size Standard v2.0 (Applied Biosystems). In case of the IGK clonality assay, PCR products were also analyzed by heteroduplex analysis and electrophoresis in non-denaturing polyacrylamide gels (HD-PAGE), stained with ethidium bromide and visualized under UV light. The HD-PAGE method discriminates amplification products according to their size and nucleotide composition, and is useful in cases with borderline (doubtful) GS results. Amplified products from diagnostic samples were interpreted according to the manufacturer’s instructions. Samples that failed to amplify following repeated testing were reported as ‘not detected’ (i.e. clonality could not be detected due to insufficient quality or quantity of DNA for analysis) and were excluded from the study.

## Results

**Figure 1:**
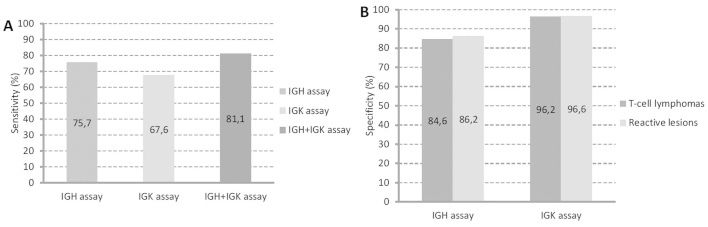
Sensitivity (A) and specificity (B) of the IGH, the IGK and combined IGH+IGK clonality assays determined in a group of B-cell lymphomas, T-cell lymphomas and reactive lesions. To determine the sensitivity and the specificity of IGH/IGK clonality assays the results of molecular testing were compared with the final diagnosis of each lymphoid proliferation. The sensitivity of each clonality assay was calculated using the equation TP/(TP+FN); TP (true positives), monoclonal (M) and ‘monoclonal in a polyclonal background’ (M/P) results of the IGH, the IGK and combined IGH+IGK clonality assay in a group of B-cell lymphomas; FN (false negatives), polyclonal (P) results of the IGH, the IGK and combined IGH+IGK clonality assay in a group of B-cell lymphomas. The specificity of the IGH and the IGK clonality assay was determined separately for T-cell lymphomas and for reactive lesions. The specificity of each assay was calculated using the equation TN/(TN+FP); TN (true negatives), polyclonal (P) results of the IGH and the IGK clonality assay in a group of T-cell lymphomas or in reactive lesions; FP (false positives), monoclonal (M) and ‘monoclonal in a polyclonal background’ (M/P) results of the IGH and the IGK clonality assay in a group of T-cell lymphomas or reactive lesions. The specificity of combined IGH+IGK assay was not determined.

Ninety-two specimens from 80 patients with the final diagnosis of mature B-cell lymphoma (37 specimens), T-cell lymphoma (26 specimens) and reactive lymphoid proliferation (29 specimens) were analyzed for B-cell clonality. According to the WHO classification, our series of B-cell lymphomas comprised 2 specimens of mantle cell lymphoma (MCL), 8 specimens of follicular lymphoma (FL), 11 specimens of nodal marginal zone B-cell lymphoma (MZL), 2 specimens of extranodal marginal zone lymphoma of mucosa-associated tissue (MALT lymphoma), 3 specimens of diffuse large B-cell lymphoma (DLBCL), 1 specimen of B-cell lymphoma with features between DLBCL and Burkitt lymphoma (BL), 1 specimen of plasmablastic lymphoma, 3 specimens of lymphoplasmacytic lymphoma and 6 specimens of unclassified B-cell lymphoma (B-cell lymphoma, unclassified). All specimens were analyzed for both, *IGH* and *IGK* clonality and detection rates of the IGH and the IGK clonality assays were compared. The results of clonality analysis using the IGH, the IGK and combined IGH+IGK clonality assays are presented in Table I. The calculated sensitivity and specificity of the IGH/IGK clonality assays are shown in Fig. 1.

### The BIOMED-2 IGH clonality assay

Monoclonal *IGH* rearrangements corresponding to monoclonal B-cell proliferations were detected in 27 of 37 B-cell lymphoma specimens (73.0%). Polyclonal *IGH* rearrangements corresponding to polyclonal B-cell proliferations were detected in 9 B-cell lymphoma specimens (24.3%). In one specimen of unclassified B-cell lymphoma monoclonal *IGH* products in a background of polyclonal products were detected and this specimen was concluded as borderline (monoclonal in a polyclonal background). Polyclonal *IGH* rearrangements were detected in MZL (4 specimens), FL (2 specimens), unclassified B-cell lymphoma (2 specimens) and B-cell lymphoma with features between DLBCL and BL (1 specimen). In 22 of 27 *IGH* monoclonal B-cell lymphoma specimens also monoclonal *IGK* rearrangements were detected. Four specimens with monoclonal *IGH* rearrangements were polyclonal by the IGK assay and one specimen with monoclonal *IGH* rearrangements was *IGK* borderline. The *IGH* borderline specimen was polyclonal by the IGK assay.

In the T-cell lymphoma group, polyclonal *IGH* rearrangements were detected in 22 of 26 analyzed specimens (84.6%) and monoclonal *IGH* rearrangements were detected in 4 specimens (15.4%). In the group of reactive specimens, polyclonal *IGH* rearrangements were detected in 25 of 29 cases (86.2%) and monoclonal *IGH* rearrangements were detected in 4 cases (13.8%).

### The BIOMED-2 IGK clonality assay

The detection rate of the BIOMED-2 IGK clonality assay was lower than that of the IGH assay. Monoclonal *IGK* rearrangements were detected in 23 of 37 analyzed B-cell lymphoma specimens (62.2%). Of these, 22 specimens were monoclonal also by the IGH assay and one specimen was IGH polyclonal. Polyclonal *IGK* rearrangements were detected in 12 specimens (32.4%). Two specimens of B-cell lymphoma were borderline for *IGK* rearrangements (monoclonal in a polyclonal background) (two BM aspirates with minimal infiltration with lymphoma cells from patients with FL and lymphoplasmacytic lymphoma, respectively). Polyclonal *IGK* rearrangements were detected in MZL (5 specimens), FL (3 specimens), unclassified B-cell lymphoma (2 specimens), DLBCL (1 specimen) and B-cell lymphoma with features between DLBCL and BL (1 specimen).

### Analysis of IGK amplification products

We also compared the efficacy of GeneScan (GS) and HD-PAGE analysis for the discrimination between monoclonal and polyclonal *IGK* rearrangements in a series of 27 B-cell lymphomas from 2011. In this regard, we evaluated only Vκ-Jκ amplification products (tube IGK-A). The results of GS and HD-PAGE analysis in a group of 27 B-cell lymphomas are shown in Table II.

Using the GS analysis we were able to discriminate between monoclonal and polyclonal Vκ-Jκ rearrangements in 26 specimens (96.3%); 13 specimens had monoclonal and 13 specimens had polyclonal Vκ-Jκ rearrangements. One specimen was borderline (monoclonal in a polyclonal background) by GS analysis, while HD-PAGE detection showed polyclonal ‘smear’ (Table II).

**Figure 2:**
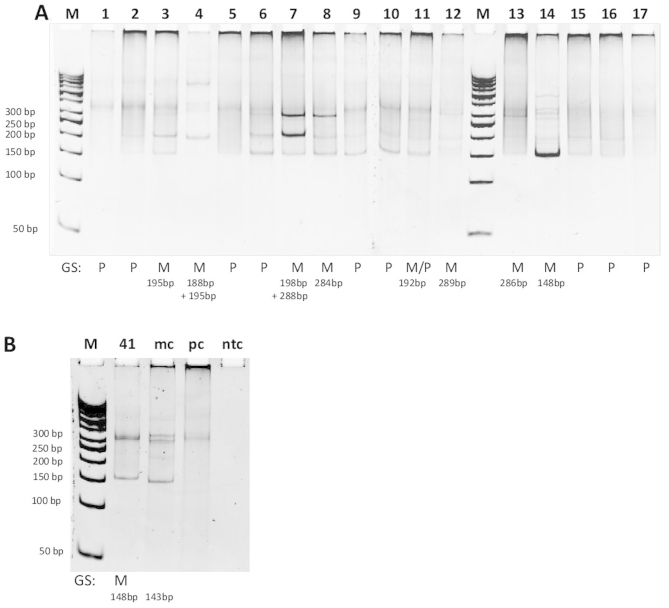
HD-PAGE detection of Vκ-Jκ rearrangements (tube IGK-A) in representative B-cell lymphoma cases. M, molecular weight marker (50 bp DNA ladder); lanes 1–17 (A) and 41 (B) represent amplifications from B-cell lymphoma cases; mc, monoclonal control; pc, polyclonal control; ntc, no template control (water instead of DNA); the presence of one or two distinct bands in the expected size range (120–160, 190–210 and 260–300 bp) indicates monoclonality; ‘smear’ in the expected size range indicates polyclonality; results of GeneScan analysis (GS) of the same cases are presented on the bottom of the gel; M, monoclonal; P, polyclonal; M/P, ‘monoclonal in a polyclonal background’. In cases 3, 4 and 13 monoclonal Vκ-Jκ amplified products were observed by GS, but after HD-PAGE analysis ‘monoclonal in a polyclonal background’ pattern was observed (A). Conversely, in cases 6, 9 and 10 polyclonal Vκ-Jκ products were detected by GS analysis, but HD-PAGE analysis showed ‘monoclonal in a polyclonal background’ pattern (A). Monoclonal Vκ-Jκ product of 148 bp detected by GS in a case of suspected IGL^+^ B-cell lymphoma was confirmed by HD-PAGE analysis (B).

The interpretation of HD-PAGE results was straightforward in 18 of 27 analyzed specimens (66.7%), in which clear monoclonal bands (6 specimens) or polyclonal ‘smears’ (12 specimens) were detected. On the contrary, 9 of 27 (33.3%) of analyzed specimens showed ‘monoclonal in a polyclonal background’ pattern by HD-PAGE analysis (6 specimens were monoclonal and 3 specimens were polyclonal by GS analysis) and were difficult to interpret (Table II). HD-PAGE detection of Vκ-Jκ amplification products in 17 representative B-cell lymphoma specimens are shown in Fig. 2A.

In routine diagnostic series, the HD-PAGE analysis was performed only in specimens with doubtful results of the GS analysis. Monoclonal Vκ-Jκ amplification product by GS analysis in the case of suspected B-cell lymphoma with the expression of λ light chains (IGL^+^ B-cell lymphoma) was confirmed by HD-PAGE method (Fig. 2B).

Among 26 analyzed T-cell lymphoma specimens, polyclonal *IGK* rearrangements were detected in 25 specimens (96.2%) and monoclonal *IGK* rearrangements in 1 specimen (3.8%). Twenty-eight of 29 reactive lymphoid proliferations were polyclonal for *IGK* rearrangements (96.5%). In one reactive specimen monoclonal *IGK* rearrangement was detected.

### The combined IGH and IGK assay

By the combination of the IGH and the IGK clonality assay, monoclonal B-cell populations were detected in 28 specimens of B-cell lymphoma (75.7%). Of these, 22 specimens were monoclonal for both the IGH and the IGK clonality. Two specimens with polyclonal Ig rearrangements detected by one of the clonality assays (IGH or IGK) and borderline result by the other assay, were concluded as borderline (monoclonal in a polyclonal background). Polyclonal rearrangements in both genes, the *IGH* and the *IGK*, were detected in 7 B-cell lymphoma specimens (18.9%). Considering monoclonal and borderline cases as ‘true positives’ the detection rate of B-cell clonality determined by the combination of IGH and IGK was 81.1% (30/37).

**Figure 3:**
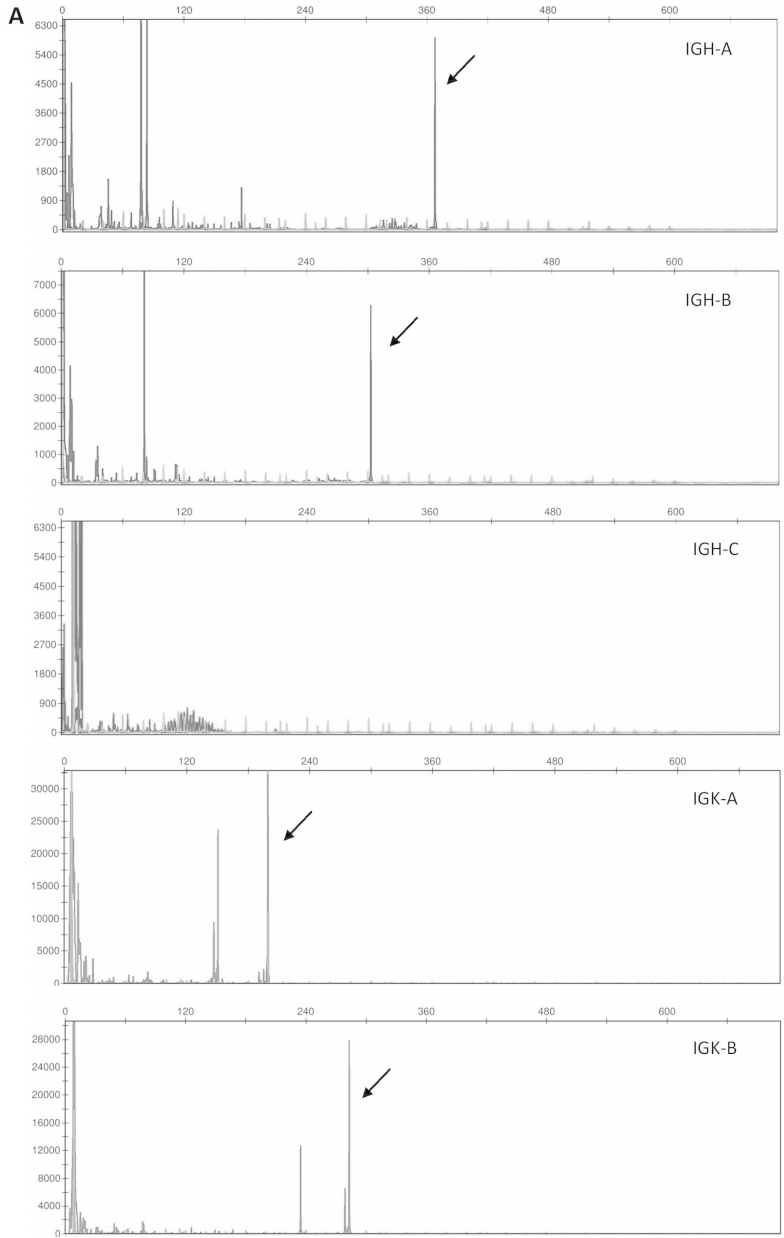

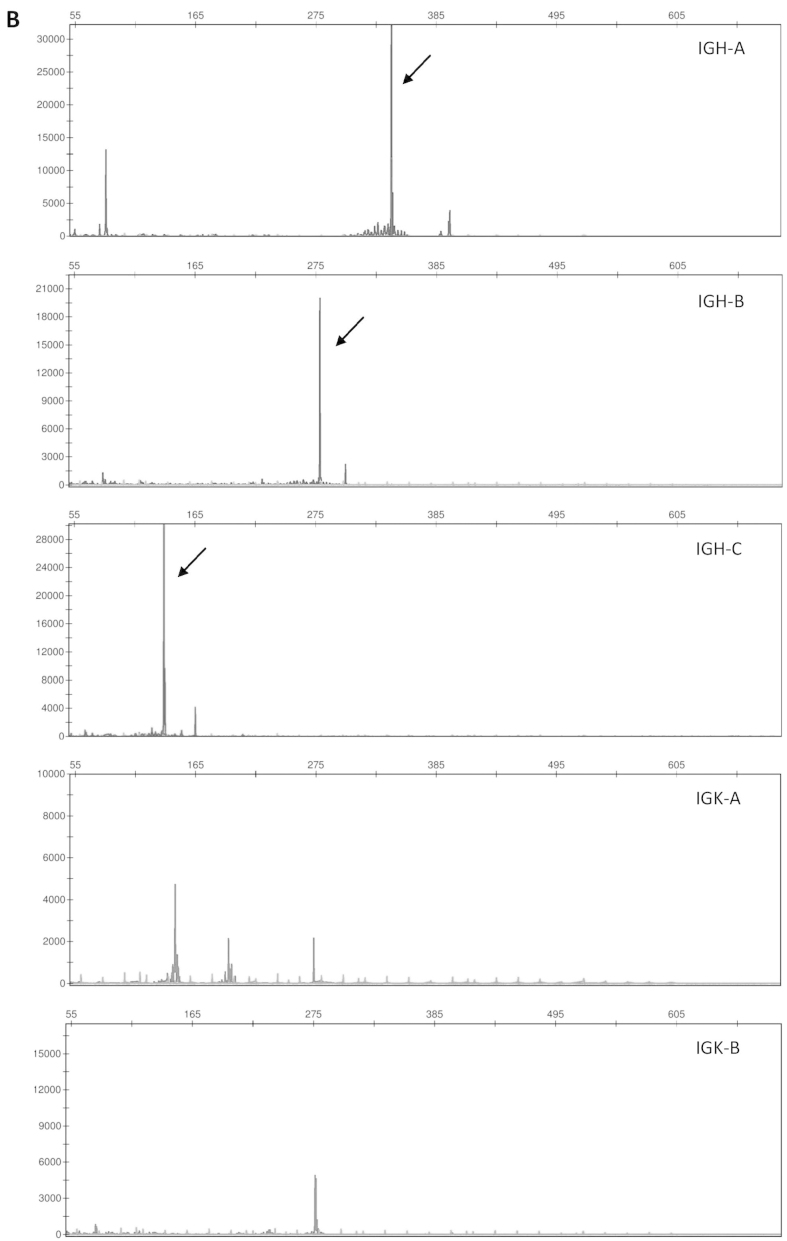

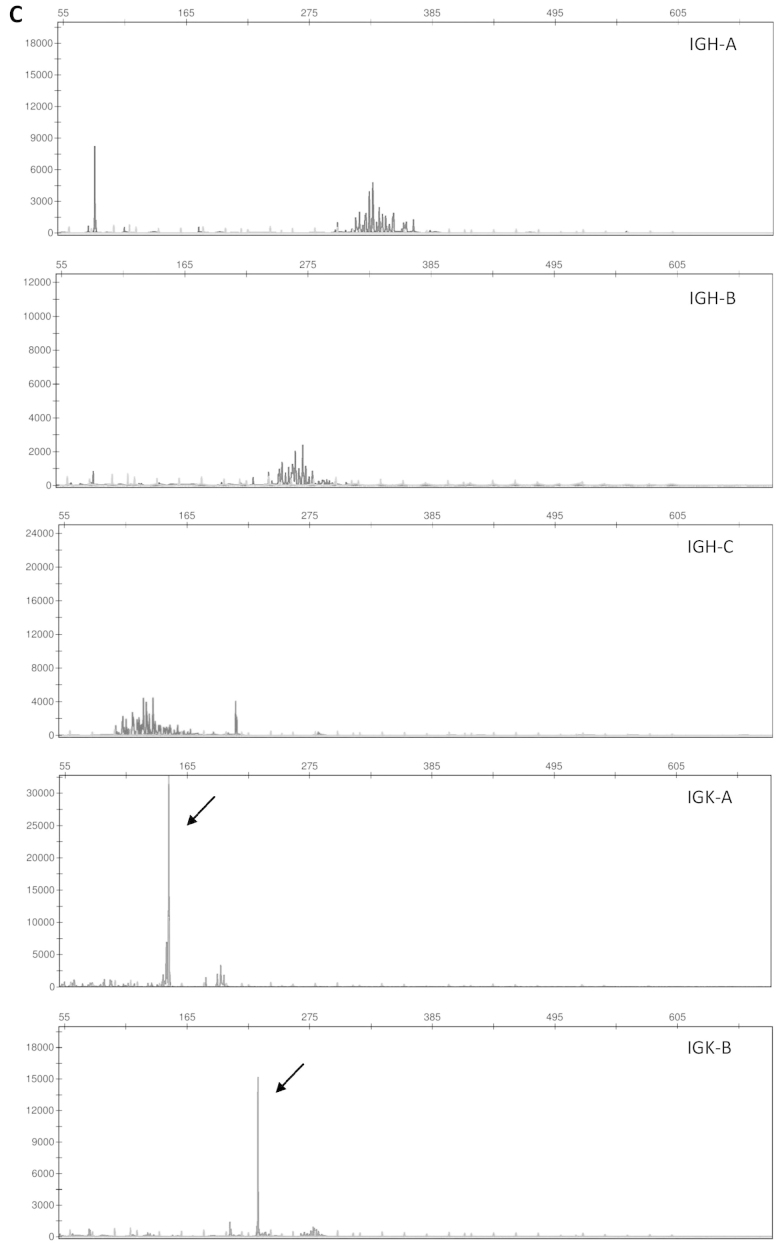
GeneScan (GS) detection of IGH and IGK amplification products in 3 representative cases of B-cell lymphoma. (A) Monoclonal rearrangements detected in both, the *IGH* and the *IGK* genes in a patient with the diagnosis of nodal marginal zone B-cell lymphoma (MZL). Arrows indicate monoclonal amplification products (peaks) of 368 bp (tube IGH-A), 303 bp (tube IGH-B), 199 bp (tube IGK-A) and 283 bp (tube IGK-B). (B) Monoclonal rearrangement in the *IGH* gene and polyclonal amplification pattern (Gaussian curve) in the *IGK* gene in a patient with the diagnosis of MZL. Arrows indicate monoclonal peaks of 344 bp (tube IGH-A), 279 bp (tube IGH-B) and 137 bp (tube IGH-C). (C) Polyclonal amplification pattern in the *IGH* gene and monoclonal rearrangements in the *IGK* gene in a patient with the diagnosis of unclassified B-cell lymphoma with the expression of the immunoglobulin λ light chains (IGL^+^ B-cell lymphoma). Arrows indicate monoclonal peaks of 148 bp (tube IGK-A) and 228 bp (tube IGK-B). Monoclonal IGK-A product of 148 bp was verified by HD-PAGE analysis (Fig. 2B).

Three representative specimens with monoclonal rearrangements in both genes, *IGH* and *IGK*, monoclonal *IGH* and polyclonal *IGK* rearrangement, and polyclonal *IGH* and monoclonal *IGK* rearrangement, respectively, are presented in Fig. 3.

## Discussion

In the present study, we evaluated the added value of standardized BIOMED-2 assay for the detection of clonal *IGK* gene rearrangements in the diagnostic setting of suspected B-cell lymphomas. The sensitivity of the IGK assay (67.6%) in our diagnostic series of B-cell lymphomas was lower than expected from the literature (6–8,17). The sensitivity of the IGH assay in our series was 75.7%, which is also lower than some of the authors reported (10,18), but higher than the obtained sensitivity of the IGK assay. With the combination of both assays (IGH+IGK) the sensitivity of the B-cell clonality detection was improved to 81.1% (Table I, Fig. 1). Nevertheless, we were not able to achieve the B-cell clonality detection rates reported in most studies using the BIOMED-2 protocols, even with the combination of the IGH and the IGK assay (6–8,17).

The lower sensitivity of the IGH assay in our series is probably related to a rather high percentage of included GC/post-GC B-cell lymphomas (29 of 37), for which it has been well documented that frequent somatic hypermutations contribute to a lower IGH monoclonality rate (6–8,18). Indeed, we detected monoclonal *IGH* rearrangements in only 22 of 29 (75.9%) GC/post-GC B-cell lymphomas (Table I), which is in agreement with other studies (54–85%) (6,7,10,14,18). Therefore, we expected a higher clonality detection rate by the IGK assay in this group of B-cell lymphomas, somewhere in the range of 74–86% (6,7,10,14,18). Surprisingly, monoclonal *IGK* rearrangements were detected in only 19 of 29 (65.5%) GC/post-GC B-cell lymphoma cases (Table I). The detection rates of the IGK assay in the most common GC/post-GC B-cell lymphoma entities, follicular lymphoma and diffuse large B-cell lymphoma were 62.5% (5 of 8) and 66.7% (2 of 3), respectively (Table I). Causes for the low sensitivity of the IGK assay in our hands, especially in histologically and immunophenotypically confirmed cases of GC/post-GC B-cell lymphoma subtypes, are not clear.

The comparison of GeneScan (GS) and heteroduplex pretreatment-polyacrylamide gel electrophoresis (HD-PAGE) methods for analysis of *IGK* gene rearrangements showed a better performance of the GS analysis in our series of 27 B-cell lymphomas analyzed by both methods. Namely, we were able to discriminate between monoclonal and polyclonal Vκ-Jκ rearrangements (tube IGK-A) in 26 cases (96.3%) using the GS analysis and in only 18 cases (66.7%) using HD-PAGE analysis (Table II). One case was borderline (monoclonal in a polyclonal background) with the GS analysis, whereas 9 cases were borderline with the HD-PAGE method. According to the EuroClonality/BIOMED-2 guidelines for interpretation and reporting of Ig/TCR clonality testing in suspected lymphoid proliferations, GS and HD-PAGE methods have complementary value for analysis of *IGK* rearrangements (19). The HD-PAGE analysis is recommended in cases with doubtful GS results, in particular when a prominent peak of ~148 bp is detected in tube IGK-A (Vκ-Jκ rearrangements). It is well known that in polyclonal samples this peak represents rearrangements of different sequences with the same size due to limited junction diversity of the rearranged *IGK* gene (19,20). Indeed, the monoclonal Vκ-Jκ product of 148 bp detected by GS analysis in a case of suspected IGL^+^ B-cell lymphoma was confirmed by the HD-PAGE method (Fig. 2B).

The specificity of the IGK assay was higher than the specificity of the IGH assay (Fig. 1). It was 96.2% in the group of mature T-cell lymphomas and 96.6% in the group of reactive lesions, which is comparable to other studies using the BIOMED-2 protocols (17,21,22). The specificity of the IGH assay in the group of T-cell lymphomas (84.6%) and in the group of reactive lesions (86.2%) is comparable to the one determined in our previous study and does not need further discussion (15).

In conclusion, the present study was applied to demonstrate the utility of combined IGH+IGK clonality assay for the assessment of B-cell clonality in suspected B-cell lymphomas with inconclusive clinical and cyto/histological diagnosis. Using the combined IGH+IGK clonality assay, the overall detection rate of B-cell clonality was increased by 5.4% (from 75.7% using only the IGH assay to 81.1% using both, IGH and IGK assays). Thus, we confirmed the added value of standardized BIOMED-2 IGK assay for the assessment of B-cell clonality. However, the sensitivity of standardized BIOMED-2 IGK assay in the present study was lower than expected, even in GC/post-GC B-cell lymphoma cases. Furthermore, care should be taken with the interpretation of the IGK amplification products due to restricted *IGK* junctional diversity.

We are aware that our conclusions derive from a rather small diagnostic series with only 37 specimens of confirmed B-cell lymphomas. Certainly, the evaluation of a larger series of B-cell lymphomas is needed for firmer conclusions. With these considerations in mind, we believe that the assessment of *IGK* gene rearrangements is valuable in demonstrating the B-cell clonality in the diagnostic setting, in particular in the absence of clonal *IGH* rearrangements.

## Figures and Tables

**Table I:** Results of clonality analysis using the IGH, IGK and combined IGH+IGK clonality assays in 92 specimens of lymphoid proliferations (LP).

Diagnosis	No. of *IGH* monoclonal/no. of tested specimens (%)	No. of *IGK* monoclonal/no. of tested specimens (%)	No. of IGH+K monoclonal/no. of tested specimens (%)
B-cell lymphoma			
MALT lymphoma	2/2 (100.0)	2/2 (100.0)	2/2 (100.0)
MCL	2/2 (100.0)	2/2 (100.0)	2/2 (100.0)
FL	6/8 (75.0)	5/8 (62.5)	7/8 (87.5)
DLBCL	3/3 (100.0)	2/3 (66.7)	3/3 (100.0)
DLBCL/BL	0/1 (0.0)	0/1 (0.0)	0/1 (0.0)
MZL	7/11 (63.6)	6/11 (54.5)	7/11 (63.6)
Plasmablastic lymphoma	1/1 (100.0)	1/1 (100.0)	1/1 (100.0)
Lymphoplasmacytic lymphoma	3/3 (100.0)	3/3 (100.0)	3/3 (100.0)
B-cell lymphoma, unclassified	4/6 (66.7)	4/6 (66.7)	5/6 (83.3)
Total B-cell lymphomas	28/37 (75.7)	25/37 (67.6)	30/37 (81.1)
T-cell lymphomas	4/26 (15.4)	1/26 (3.8)	4/26 (15.4)
Reactive specimens	4/29 (13.8)	1/29 (3.4)	5/29 (17.3)
Total LP	36/92 (39.1)	27/92 (29.3)	39/92 (42.4)

Borderline cases (monoclonal in a polyclonal background) were considered as monoclonal. MALT lymphoma, extranodal marginal zone lymphoma of mucosa-associated tissue; MCL, mantle cell lymphoma; FL, follicular lymphoma; DLBCL; diffuse large B-cell lymphoma; DLBCL/BL, B-cell lymphoma with features between DLBCL and Burkitt lymphoma; MZL, marginal zone B-cell lymphoma; LP, lymphoid proliferation.

**Table II:** Comparison of GeneScan (GS) and heteroduplex pretreatment-polyacrylamide gel electrophoresis (HD-PAGE) methods for analysis of *IGK* gene rearrangements in a group of 27 B-cell lymphomas.

A, The results of GS and HD-PAGE analysis in 27 cases of B-cell lymphomas						

Sample no.	Diagnosis	IGK-A-GS	IGK-A-HD	IGK-A-final	IGK-B	IGK-final
1	MZL	P	P	P	P	P
2	DLBCL/BL-like	P	P	P	P	P
3	MZL	M	M/P	M	P	M
4	Lymphoplasmacytic	M	M/P	M	M	M
5	FL	P	P	P	P	P
6	Lymphoplasmacytic	P	M/P	M/P	M/P	M/P
7	Lymphoplasmacytic	M	M	M	M	M
8	FL	M	M	M	M	M
9	MZL	P	M/P	M/P	M	M
10	MCL	P	M/P	M/P	M	M
11	FL	M/P	P	M/P	P	M/P
12	MZL	M	P	M	P	M
13	MZL	M	M/P	M	P	M
14	Plasmablastic	M	M	M	M	M
15	B-cell lymphoma, unspecified	P	P	P	P	P
16	DLBCL	P	P	P	P	P
17	FL	P	P	P	M	M
18	MALT lymphoma	M	M/P	M	P	M
19	MZL	P	P	P	P	P
20	B-cell lymphoma, unspecified	M	M/P	M	P	M
21	MZL	P	P	P	P	P
22	MZL	M	M/P	M	P	M
23	DLBCL	M	M	M	M	M
24	DLBCL	M	M	M	M	M
25	B-cell lymphoma, unspecified	M	M	M	P	M
26	FL	P	P	P	P	P
27	FL	P	P	P	ND	P

B, The summary of results obtained by GS and HD-PAGE analysis of tube A (Vκ-Jκ) amplification products in 27 cases of B-cell lymphomas						

Summary						Data

No. of IGK monoclonal by both methods/no. of tested specimens (%)						6/27 (22.2)
No. of IGK polyclonal by both methods/no. of tested specimens (%)						10/27 (37.0)
No. of IGK borderline by both methods/no. of tested specimens (%)						0/27 (0.0)
No. of discordant results between GS and HD-PAGE methods/no. of tested specimens (%)						11/27 (40.7)

MZL, marginal zone B-cell lymphoma; DLBCL/BL-like, B-cell lymphoma with features between DLBCL and Burkitt lymphoma; FL, follicular lymphoma; MCL, mantle cell lymphoma; DLBCL, diffuse large B-cell lymphoma; MALT lymphoma, extranodal marginal zone lymphoma of mucosa-associated tissue. IGK-A-GS, tube IGK-A (Vκ-Jκ) amplification products analyzed by GS method; IGK-A-HD, tube IGK-A amplification products analyzed by HD-PAGE method; IGK-A-final, final results (conclusions) for tube IGK-A amplification products; IGK-B, tube IGK-B (κ deleting element-Kde) amplification products (analyzed only by GS method); IGK-final, final results (conclusions) for IGK rearrangements. M, monoclonal; P, polyclonal; M/P, ‘monoclonal in a polyclonal background’ (borderline); ND, not detected.
